# Integrated, Speckle-Based Displacement Measurement for Lateral Scanning White Light Interferometry

**DOI:** 10.3390/s21072486

**Published:** 2021-04-02

**Authors:** Gert Behrends, Dirk Stöbener, Andreas Fischer

**Affiliations:** 1 Bremen Institute for Metrology, Automation and Quality Science, University of Bremen, 28359 Bremen, Germany; d.stoebener@bimaq.de (D.S.); andreas.fischer@bimaq.de (A.F.); 2 MAPEX Center for Materials and Processes, 330440, University of Bremen, 28334 Bremen, Germany

**Keywords:** white light interferometry, digital speckle correlation, surface topography, displacement measurement

## Abstract

Lateral scanning white light interferometry (LSWLI) is a promising technique for high-resolution topography measurements on moving surfaces. To achieve resolutions typically associated with white light interferometry, accurate information on the lateral displacement of the measured surface is essential. Since the uncertainty requirement for a respective displacement measurement is currently not known, Monte Carlo simulations of LSWLI measurements are carried out at first to assess the impact of the displacement uncertainty on the topography measurement. The simulation shows that the uncertainty of the displacement measurement has a larger influence on the total height uncertainty than the uncertainty of the displacing motion itself. Secondly, a sufficiently precise displacement measurement by means of digital speckle correlation (DSC) is proposed that is fully integrated into the field of view of the interferometer. In contrast to externally applied displacement measurement systems, the integrated combination of DSC with LSWLI needs no synchronization and calibration, and it is applicable for translatory as well as rotatory scans. To demonstrate the findings, an LSWLI setup with integrated DSC measurements is realized and tested on a rotating cylindrical object with a surface made of a linear encoder strip.

## 1. Introduction

### 1.1. Motivation

Rising demands regarding the quality of optically smooth surfaces of consumer goods and industrial intermediate products necessitate metrology that is able to quantify the topography of these surfaces in a quick and accurate manner. Systems capable of in-process measurements are especially interesting for manufacturers, as early detection of defects reduces production costs [1,2]. For delicate surfaces, such as optical components or highly reflective functional and decorative surfaces, a contactless method for topography measurement is desired. In addition, many manufacturing processes involve continuously moving, rotating materials or tools, such as the rolling of sheet metal. Therefore, a strong demand for precise topography measurements on curved surfaces of continuously rotating objects exists.

### 1.2. State of the Art

Due to its areal measurement capabilities and low measurement uncertainties of <1 nm, white light interferometry (WLI) has become one of the standard techniques for topography measurement. The term “white light interferometry” itself was coined in the 1970s by Fluornoy et al., who applied the principle in film thickness gauging [3]. WLI or coherence scanning interferometry, as it is referred to in DIN EN ISO 25178 [4], first appeared in its today most commonly implemented form as vertical scanning white light interferometry (VSWLI) in the late 1980s [5,6,7]. Comprehensive information on VSWLI can be found in the review papers of de Groot and Wyant [8,9]. 

VSWLI requires the objects to stand still during vertical scanning. Therefore, it is not usable for measurements on continuously moving objects, such as the rollers for sheet metal production. For such measurements, lateral scanning white light interferometry (LSWLI) can be used. LSWLI is a variant of white light interferometry that was first described by Olszak [10]. It uses a straight or curved scan path to record lateral and axial spatial information in one single motion. The consequence of the lateral scanning is that LSWLI requires a relative lateral movement between optics and object. This puts LSWLI at an advantage for in-process measurements on continuously moving objects.

LSWLI was originally applied on planar objects with straight, translatory scan motions [10]. To enable the measurement of cylindrical rollers for sheet metal production, LSWLI has recently been advanced to also work for rotatory scanning motions by taking the curvature of the scan path into account for the topography calculation [11].

The topographical height $h_{12}$ between two surface points i=1,2 on a translatory scan path can be calculated by using the lateral positions $x_{i}$ in the field of view, at which each point intersects the plane of zero optical path length difference between the light paths of the interferometer, and the common tilt angle Θ of the surface points’ scan paths:

(1) $$h_{12}=\left(x_{1}-x_{2}\;\right)\cdot \sin\left(\Theta \right)$$

Both terms—the lateral positions $x_{i}$ and the tilt of the scan path Θ—are extractable from the scan’s interference signal, the so-called correlogram. The lateral positions $x_{i}$ correspond to the maxima positions of the correlogram’s envelopes, and the tilt angles Θ to its fringe frequency, as demonstrated by Munteanu [12]. As recently shown by Behrends et al. in [11], the height equation for rotatory scan paths becomes:

(2) $$h_{12}=\frac{\left(x_{1}-x_{2}\right)\tan\left(\frac{\Theta_{1}+\Theta_{2}}{2}\right)}{\cos\left(\Theta_{1}\right)-\cos\left(\Theta_{2}\right)}\;\cdot \left(\cos\left(\Theta_{1}\right)\sqrt{\frac{\sin^{2}\left(\Theta_{2}\right)}{\cos^{2}\left(\Theta_{1}\right)}+1}-1\right)$$

where the trigonometric properties of the rotatory (circular) scan path have been incorporated into the equation by considering the changing local tangent surface angles $\Theta_{1}$ and $\Theta_{2}$ of the two surface points at the positions $x_{1}$ and $x_{2}$ to be compared. 

**Figure 1:**
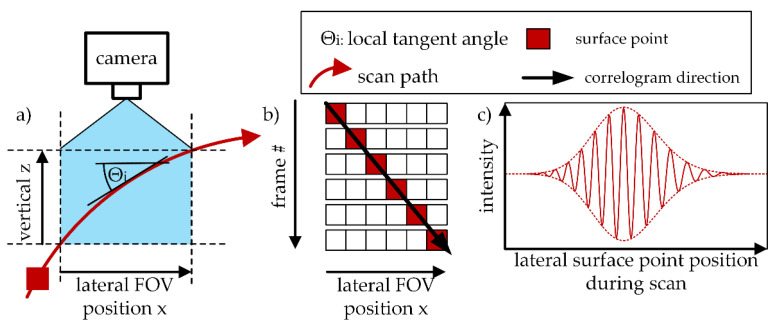
Correlogram recording in rotatory lateral scanning white light interferometry (LSWLI). (**a**) x-z orientation of the scan path in the field of view (FOV) of the camera. (**b**) Surface position in each frame of a LSWLI image series. (**c**) Correlogram of the observed surface point.

In both translatory and rotatory LSWLI, all quantities needed for the height calculation are extracted from the correlogram. Therefore, the uncertainty of $h_{12}$ depends strongly on the accuracy of the correlogram reconstruction. Figure 1 depicts the recording and correlogram reconstruction process for rotatory LSWLI. The correlograms of the surface points are reconstructed from an image series, which is recorded with a known temporal frequency. What is unknown, however, is the displacement of the observed surface between the images according to the lateral movement of the object surface through the camera’s field of view (FOV). Since the lateral displacement is crucial for the correlogram reconstruction, an accurate tracking of the surface movement during the measurement process is necessary.

A fundamental design requirement for a displacement measurement system for (translatory and rotatory) LSWLI is the necessary displacement accuracy to reconstruct the correlograms from the recordings and to enable surface topography measurements with a minimal measurement uncertainty. Considering the ideal case, the effect of the displacement measurement uncertainty on the total topography measurement uncertainty should be negligible in comparison with other uncertainty components. While the VSWLI technology can be assumed to operate close to the ideal case, the influence of displacement is considerably larger in LSWLI. This is the price for the LSWLI’s advantageous capability of measuring on surfaces in motion. For this article, the limit for the disadvantage of the real LSWLI due to this position uncertainty is based on internal project requirements, stating the height uncertainties should not be higher than twice the height uncertainty for an ideally scanning (LS)WLI without positioning uncertainty, but with the same recording and evaluation methods. Another design requirement concerns the combination of the displacement measurement system and the LSWLI in conjunction with the moving object surface. In order to be flexibly usable in different applications, the displacement measurement system should be independent from the data provided by the movement stage itself. Finally, a displacement measurement is desirable that works on both translatory and rotatory scan motions.

The importance of accurate positioning has been recognized since the inception of LSWLI. Olszak [10] noticed washing out of contours due to deviations in scanning speed, which was assumed to be at a constant speed of 1 pixel per frame. Munteanu [12] used a translation stage with an accuracy of ± 0.2 µm/mm, which was also set to a scanning speed of 1 pixel per frame. With this setup, a height uncertainty of 40 nm on a 8.69 µm high step height was achieved. Vibrations in the translation stage were claimed as a main contributor to the uncertainty. Guo et al. [13] combined an LSWLI-based measurement system with a nano-measuring-machine with an accuracy of 0.1 nm that tracked the position of the scanned object along the x- and *z*-axis. The scanning speed was set to 1–4 pixels per frame. Behrends et al. [11] used rotatory LSWLI with a circumferential displacement of 1 pixel per frame, which was assumed to be constant.

As a result, the positioning uncertainty of the movement stages in the reported studies, if mentioned, ranges from several hundred down to a tenth of a nanometer. However, a quantitative assessment of the influence of the positioning uncertainty on the topography height uncertainty is missing. The displacement of the object is either assumed with a constant rate or is measured with additional external sensors on the movement stage. Therefore, the displacement measurement is currently not independent from the movement stage. In particular, all realized displacement measurement systems are for translatory LSWLI and are not transferable to rotatory LSWLI. A universal displacement measurement system for both translatory and rotatory LSWLI is missing.

### 1.3. Aim and Structure

The aim is to propose an integrated displacement measurement system for LSWLI that enables precise topography measurements on continuously rotating objects. At first, the influence of the measurement uncertainty of a displacement measurement system on the topography measurement uncertainty is determined. Then, a displacement measurement with sufficient precision is realized by means of digital speckle correlation (DSC), which is integrated in a dedicated region of interest of the same camera that records the WLI signal. The displacement measurement works completely independent from the control of the rotational movement, is in perfect sync and alignment with the WLI signal, and does not require any unit conversions or calibrations to enable a precise reconstruction of the correlogram. Note that the approach is applicable for rotatory and translatory LSWLI topography measurements alike.

The measurement principle of the merged measurement system is introduced at the beginning of Section 2. The principles of both the DSC and the LSWLI sub-system are also explained briefly, including the used signal evaluation methods. Furthermore, a Monte Carlo simulation for the measurement chain is described to assess the uncertainty of the topography measurement resulting from the displacement measurement uncertainty and the noise in the interferometer signal. In the last part of Section 2, the considered experimental setup is explained. The results of the Monte Carlo simulation and an experimental validation with rotatory LSWLI measurements on a cylindrical test object are presented and discussed in Section 3. The conclusions of the article are presented together with an outlook in Section 4.

## 2. Materials and Methods

### 2.1. Measurement Principles

The measurement principle of the whole system is based on a DSC displacement measurement system integrated into the optics of an LSWLI setup by dividing the camera’s field of view into two regions of interests (ROI), a smaller one for DSC, and a larger one for WLI. Integrating both systems into one optical path and using the same camera chip for signal recording almost eliminates the influence of timing and deviations caused by sudden accelerations, as displacement and topography are measured synchronously. Using the same sensor also eliminates alignment issues between the LSWLI and the displacement measurement, as both are recorded on the same pixel grid. A third advantage of using the same sensor for both measurements is that the displacement is measured in the unit ‘pixels’, which are of the same scale as the WLI data. There is no need to convert or calibrate the systems in order to work together, which makes the whole system a flexible topography measurement system for both translatory and rotatory moving objects.

**Figure 2:**
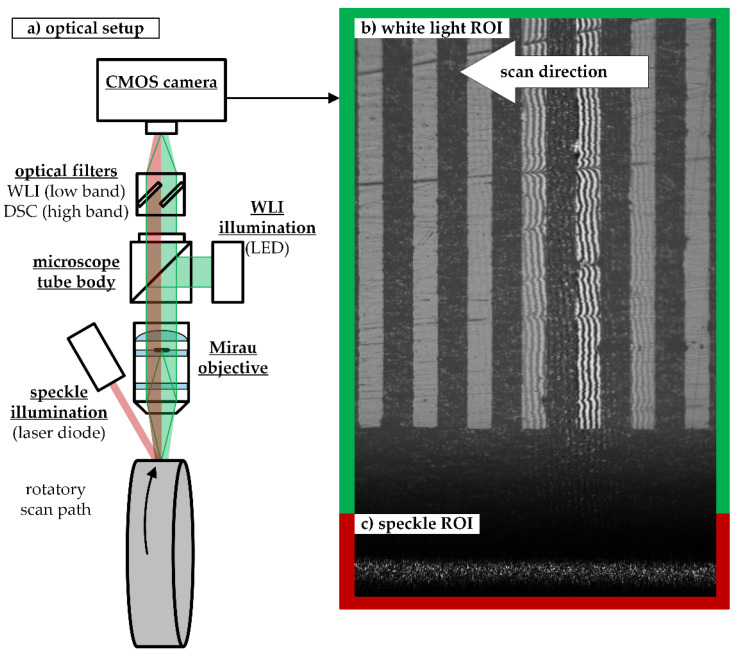
(**a**) Schematic illustration of the combined digital speckle correlation (DSC) and white light interferometry (WLI) measurement system. Green: light path for white light interferometry (WLI). Red: light path for digital speckle correlation (DSC). Right side: raw image showing both regions of interest (ROI) in the same field of view. (**b**) White light ROI. (**c**) Speckle ROI. The laser illumination profile is shaped with line optics to fit into the DSC ROI.

A schematic sketch of the whole measurement system is illustrated in Figure 2. The optical setup is based on a standard WLI, with a light emitting diode as light source and a Mirau objective creating the interference effect. The ROI for the DSC measurement is illuminated with an infrared laser diode. Both ROIs are captured with one digital camera with a global shutter. In order to optically separate the scattered light from both light sources, optical filters are inserted in the light path before the camera.

#### 2.1.1. Digital Speckle Correlation (DSC)

After recording a scan, the first step of the signal processing is determining the object displacement between consecutive frames. This information is needed to reconstruct the correlograms of the moving surface points from the image series. For this purpose, one section of the camera sensor is used for displacement measurements by means of digital speckle correlation (DSC), see Figure 2c, which enables robust, highly precise displacement measurements [14,15].

The underlying principle of DSC is digital image correlation. Assuming a rigid surface, the translatory shift between two images can be determined by finding the maximum of the cross-correlation function. The image correlation algorithm used for this article is ‘efficient subpixel registration by cross correlation’ written and published by Guizar-Sicairos et al. [16]. This algorithm is optimized regarding the computation time and memory usage, and it is able to evaluate the displacement with subpixel resolution. According to Zhang et al. [17], digital image correlation will succeed as long as the displacement is smaller than the evaluation window. The complete DSC ROI is used here as an evaluation window, which means the DSC system will not restrict the measurement capabilities, as its displacement measurement range is larger than the largest surface displacements with which LSWLI is still possible.

Digital image correlation works best with images that contain clearly defined features. In the field of surface topography, these features could be for example scratches, tool marks or intentional marks or edges. The measurement system in this article is intended to be applicable on smooth and thus practically featureless surfaces with a surface roughness in the range of 2 nm < Sq <10 nm. To ensure that the displacement measurement works regardless of the surface texture or the WLI light signal, the white light in the DSC–ROI is blocked using an optical filter in front of the camera and the image correlation is conducted on an infrared-laser-illuminated surface image, which contains speckles. Speckles appear in the observer plane due to interference of laser rays, which were reflected by a surface with nano-scale roughness. A central requirement is that the change of the evaluated speckle pattern in the two successive images remains negligibly small. According to Goodman [18], the average size of speckles depends on the aperture of the optics. While the axial size is proportional to $NA^{-2}$, the lateral size is proportional to $NA^{-1}$, meaning the speckle is much larger axially than laterally. In a typical LSWLI setup, the lateral movement of the surface is usually more than 50× larger than the axial height change due to the tilted scan path. Therefore, the DSC displacement measurement method for the intended application in this article is not restricted by changes of the speckle pattern.

#### 2.1.2. White Light Interferometry (WLI)

**Figure 3:**
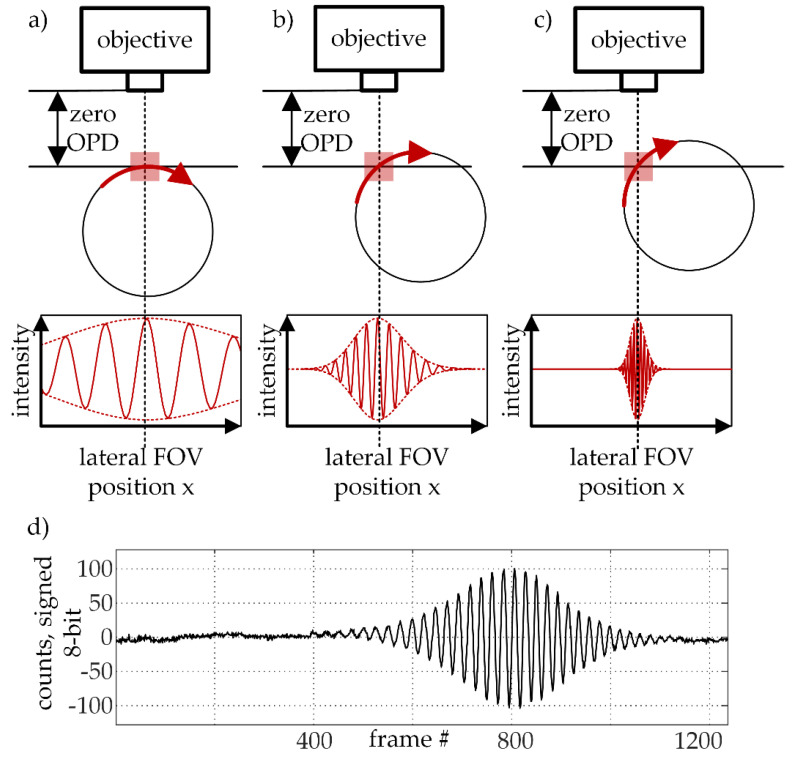
Schematic illustration of the effect of the white light interferometry (WLI) position relative to the scan path on the correlogram. The interference maximum occurs at zero optical path difference (OPD). (**a**) A shallow scan path over the apex yields a wide correlogram. The frequency of the fringes is low and the interference maximum is not as defined as in b). (**b**) A moderately tilted scan path leads to a correlogram with clear interference maximum and clear changes in intensity. (**c**) A steep scan path results in a prominently peaking interference maximum with a comparably high frequency. (**d**) Example of a real correlogram recorded with the experimental rotatory LSWLI setup.

The larger of the two ROIs on the specimen’s surface is reserved for the LSWLI topography measurement of the specimen, see Figure 2b. A crucial aspect of the WLI setup is the position and the orientation of the WLI sensor in relation to the scan path. This aspect is illustrated in Figure 3.

The width of the interference fringe structure (correlogram) in the field of view depends on the maximum height difference observed over the complete scan path for each surface element. The fringes appear in a fixed distance range from the objective and are centered around the zero-th order fringe, which marks the zero optical path length difference of the Mirau objective. The (vertical) distance range of the fringes depends on the coherence length of the used white light. For a circular, rotatory scan path this means that the fringes would appear to be broader the closer the WLI is positioned to the apex of the scan path. The apex of the scan path is here defined as the position, where the scan path is perpendicular to the observation direction of the WLI optics. A consequence of the fixed vertical range given by the fringes in combination with the lateral scanning motion is that a compromise between measurement range and measurement resolution must be made: On the one hand, measuring close to the apex enables a finer sampling of the correlogram in terms of pixels per fringe, because of the broader fringes (see Figure 3a). On the other hand, measuring further from the apex extends the measurement range since the vertical change of the scan path within the field of view is larger (see Figure 3c). In practice, an interference region spanning about one third to a half of the field of view in scan direction (Figure 3d) has been proven to be a well-fitting compromise between range and resolution for most measurement tasks. Also, to avoid additional post-measurement image rotation operations, the specimen should move as parallel to the sensor’s pixel rows as possible.

To obtain the topographical height $h_{12}$ between two surface points 1 and 2, two quantities per point need to be extracted from the correlogram: the local surface tangent angle and the position of the maximum of the envelope, see. Equation (2). Point 1 is generally chosen as the first measurement point without limiting generality.

The local surface tangent angle can be calculated from the frequencies of the correlogram using Munteanu’s method [12], as demonstrated by [11]. The frequency is evaluated using a continuous wavelet transform with Morse wavelets. The used Matlab (The MathWorks, Natick, MA, USA) implementation of the continuous wavelet transform is based on Olhede and Walden’s work on generalized Morse wavelets [19].

The position of the maximum of the correlogram’s envelope is determined in two steps. First, the envelope is calculated by applying a moving root mean square (rms) averaging algorithm on the raw correlogram. In the next step, the position of the envelope’s maximum is determined with a Gaussian fit. For the present article, the standard nonlinear least squares approach of Matlab’s fitting toolbox is applied.

#### 2.1.3. Experimental Setup

The experimental setup is realized according to the principle shown in Figure 2a. A light emitting diode with a central wavelength of 520 nm is used as the illumination source for the WLI. The speckle illumination is achieved by directing an 850 nm laser diode with a line generating lens at the DSC region of interest. The scattered light from the surface is imaged through a 10×/0.3 Mirau objective on a 2.3 MP CMOS camera. The camera is equipped with a Sony IMX174 sensor, which has a well depth of 32,406 e^−^ sampled at 8 bit. The quantum efficiency is rated at 76% at 525 nm wavelength. The surface observed by a camera pixel under the total magnification factor of the setup was determined to be 0.557 µm/pixel. All pixel values stated in this article can be converted using this conversion factor. The captured surface is divided into two ROIs by two optical filters in front of the camera. The speckles are removed from the WLI-ROI with a short-pass filter with a cut-off wave length of 750 nm. As the interference fringes are disrupting the speckle pattern, the fringes are removed from the DSC ROI using a 650 nm long-pass filter. The WLI-ROI occupies an area of $1620\;\times \;1200\;\mathrm{pixels}\;=\;902.34\;\times \;668.4{\;\text{µ}m}^{2}$ on the camera chip and the DSC-ROI occupies the remaining 300 × 1200 pixels= 167.1µm × 668.4 µm of sensor area.

The measurement object is moved by a rotation stage with a rated unidirectional repeatability of $u(\alpha_{\mathrm{motion},\mathrm{stage}})\;=\;\pm \;3.5\;\text{µ}\mathrm{rad}$. To ensure the highest possible correlogram sampling density, the stage is set to a constant rotation speed that is equivalent to an apparent surface speed of one pixel per frame. The image recording is triggered using the serial connection of the rotation stage for relaying the trigger signal sent by a Matlab script.

**Figure 4:**
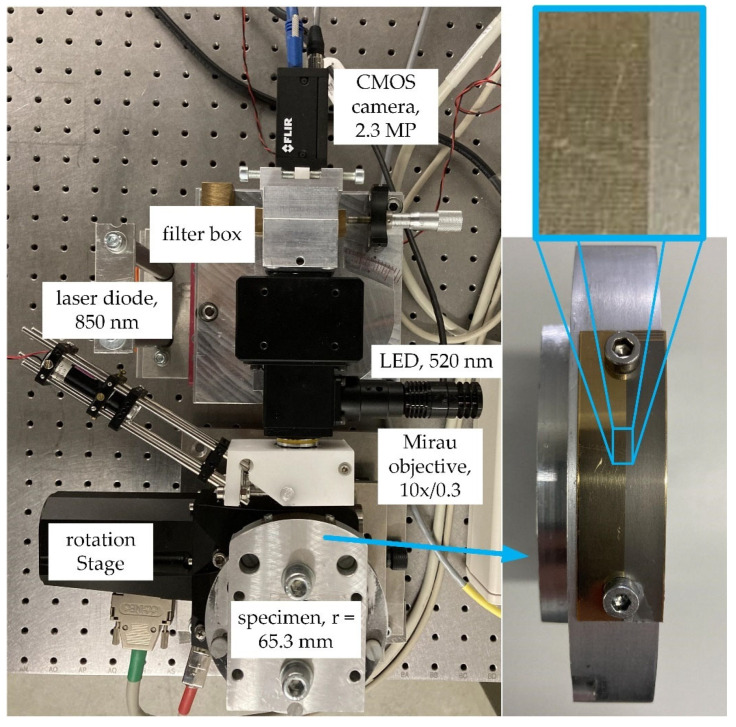
Experimental setup and measurement object. Left: Experimental setup for rotatory LSWLI. Right: Face view of the measurement object: linear encoder strip spanned on a milled base resulting in a 65.3 mm surface radius.

The measurement object for experimental validation is a section of a linear encoder strip that has been fastened on a cylinder section resulting in a surface radius of 65.3 mm. The topography has been referenced with a commercial VSWLI. The VSWLI used is from the company GBS mbH, Ilmenau, Germany, of the type smart WLI with a 10×/0.3 and a 50×/0.55 Mirau objective. With its 0.5× tube and the 50× objective it captures an area of $365.7\;\times \;228.5{\;\text{µ}m}^{2}$ per FOV and 1797.2 × 1122.9 µm^2^ with the 10× objective. It has a vertical scanning range of 400 µm. The linear encoder strip’s surface consists of alternating smooth and rough regions, manufactured by etching. The encoder strip is chosen, because its smooth regions are similar to the surfaces; the LSWLI is intended to be used for, e.g., the rolled sheet metal and the rollers mentioned in Section 1. The alternation between smooth and rough regions allows for easy visual confirmation and that lateral displacement is correctly measured and used in evaluation. Based on the object’s radius, the rotation stage’s angular positioning uncertainty can be converted to pixels, yielding $u\left(x_{\mathrm{motion},\mathrm{stage}}\right)\;=\;\pm 0.065\;\mathrm{pixels}.$ Experimental setup and the measurement object are depicted in Figure 4. It is a direct realization of the schematic setup illustrated in Figure 2a.

### 2.2. Uncertainty of Rotatory Lateral Scanning White Light Interferometry

Uncertainty propagation of h_12_

The uncertainty of the height $h_{12}$ as derived from Equation (2) for rotatory LSWLI measurements and including the covariances between $x_{i}$ and $\Theta_{i}$ reads

(3) $$u\left(h_{12}\right)=\sqrt{\begin{array}{c} \mathrm{var}\left(x_{1}\right)+\mathrm{var}\left(x_{2}\right)+\mathrm{var}\left(\Theta_{1}\right)+\mathrm{var}\left(\Theta_{2}\right)+\\ 2\cdot (\mathrm{cov}\left(x_{1},x_{2}\right)+\mathrm{cov}\left(\Theta_{1},\Theta_{2}\right)\\ +\mathrm{cov}\left(x_{1},\Theta_{1}\right)+\mathrm{cov}\left(x_{1},\Theta_{2}\right)\\ +\mathrm{cov}\left(x_{2},\Theta_{1}\right)+\mathrm{cov}\left(x_{2},\Theta_{2}\right)) \end{array}}$$

Since the covariance terms cannot be assumed as zero, because both quantities are obtained from the same correlogram signals, the uncertainty $u\left(h_{12}\right)$ is not estimated analytically or semi-analytically by using Equation (3) but numerically with a Monte Carlo simulation of the signal processing chain using synthetic correlograms.

Signal model

The core of the Monte Carlo simulation for the estimation of $u\left(h_{12}\right)$ is an idealized correlogram signal model including the sources of uncertainty. The correlogram signal model is the undisturbed light intensity $I\left(z_{j}\right)$ following the known model for WLI [20]

(4) $$I\left(z_{j}\right)=I_{0}\cdot \;\left[\;1+\exp\left[\;-4\cdot {\left(\frac{\left(z_{j}-z_{\mathrm{ref}}\right)}{l_{c}}\right)}^{2}\right]\cdot \cos\left(\frac{4\pi \left(z_{j}-z_{\mathrm{ref}}\right)}{\lambda_{0}}\right)\right]$$

where $l_{c}$ is the coherence length, $\lambda_{0}$ is the central wavelength of the white light illumination, and $z_{\mathrm{ref}}$ is the height, at which the optical path length difference is zero. In rotatory LSWLI, the vertical coordinates $z_{j}$ change during the lateral scan, which follows a curved path and therefore combines the movements in x- and z-direction. For a circularly curved scan path, the geometric relationship is

(5) $$z_{j}-z_{\mathrm{ref}}=x_{j}\cdot \tan\left(\Theta_{j}\right)-x_{\mathrm{ref}}\cdot \tan\left(\Theta_{\mathrm{ref}}\right)$$

and every $x_{j}$, j = 1,…, $n_{\mathrm{corr}}$, represents one position in the correlogram. While the correlogram positions $x_{j}$ are confined to the coordinate systems of the field of view, the camera itself has its own offset position with respect to the apex of the rotatory scan path, which is taken into account by simple addition of the offset $x_{\mathrm{offset}}$ to the x-coordinates of the correlogram, yielding the x-coordinate of the scan path $x_{j}^{\prime }=x_{j}+x_{\mathrm{offset}}$. The z-coordinates of the simulated cylinder observed in the field of view are calculated from the absolute coordinates $x_{j}^{\prime }$ of the scan path and the radius r using the geometric relationship $z_{j}=\;r\;-\;\sqrt{r^{2}-{x_{j}^{\prime }}^{2}}$.

Firstly, to include apparent irregularities of the scan motion, e.g., due to a jittery rotation stage or due to fluctuations in the triggering of the camera, each $x_{j}$ is superposed with additive white Gaussian noise that has a mean value of zero and the standard deviation $u\left(x_{\mathrm{motion}}\right)$. With the resulting x-positions, the intensity signal is calculated according to Equations (4) and (5). As the second source of uncertainty, the image noise originating from the camera and natural light fluctuations are modeled by superposing the calculated intensity signal $I\left(z_{j}\right)$ with additive white Gaussian noise that has a zero mean and a standard deviation u(I). Thirdly, the uncertainty $u\left(x_{\mathrm{measurement}}\right)$ of the displacement measurement is included by drawing the intensity values of the correlogram signal at random positions with the mean value $x_{j}$ and the standard deviation $u\left(x_{\mathrm{measurement}}\right)$ using a Gaussian distribution. Finally, the effect of the sampling resolution due to the pixel grid of the camera is realized by rounding the sampling positions $x_{j}$ to full pixels.

The resulting correlogram, which now contains the uncertainty contributions u(I) from the image noise, $u\left(x_{\mathrm{motion}}\right)$ from the positioning and $u\left(x_{\mathrm{measurement}}\right)$ from the displacement measurement, is ready for the subsequent signal evaluation in the Monte Carlo simulation.

Note further assumptions that are included in the signal model:

- In the lateral direction, the influence of the varying surface gradient due to the curvature of the scan path on the imaging of the surface onto the camera pixels is negligible. Example: If the surface is tilted 0° at one edge of the sensor and 5° on the other, the pixel at the 5° edge records 100.38 % of the area captured at the 0° edge.
- The change in intensity due to Lambertian reflection at different angles is deemed insignificant for the observed angle range.
- The surface moves strictly in x-direction. There is no movement in y-direction, perpendicular to the scan direction.
- The ideal correlograms are always centered in the field of view.

Monte Carlo simulation setup

During the Monte Carlo simulation, synthetic correlograms with preset uncertainty terms are calculated and evaluated by the signal processing methods described in Section 2.1.2. The simulated object is an ideal cylinder with a radius of r = 65.3 mm, which is equal to the object’s radius used in the experiment. The correlograms are synthesized with a maximum amplitude of 80 counts, mimicking the experimental setup. The added intensity noise has an uncertainty of u(I) = 4 counts, which is also comparable to the noise levels observed in the experiment. The simulated sampling of the correlogram is carried out with a resolution of 1 pixel. The simulation is set to run 5000 repetitions with randomized uncertainty terms with a range of $u\left(x_{\mathrm{motion}}\right)\;=\;0.1\;\ldots \;0.4\;\mathrm{pixel}$ and $u\left(x_{\mathrm{measurement}}\right)\;=\;0.02\;\ldots \;0.05\;\mathrm{pixel}$, each with the other uncertainty term set to zero. The physical size of a pixel is set to 0.557 µm, based on the camera and magnification of the experimental setup. Each combination of uncertainties was simulated for 14 apex distances ranging from 0.1–2.5 mm with regard to the edge of the field of view closest to the apex.

## 3. Results

### 3.1. Monte Carlo Simulation

**Figure 5:**
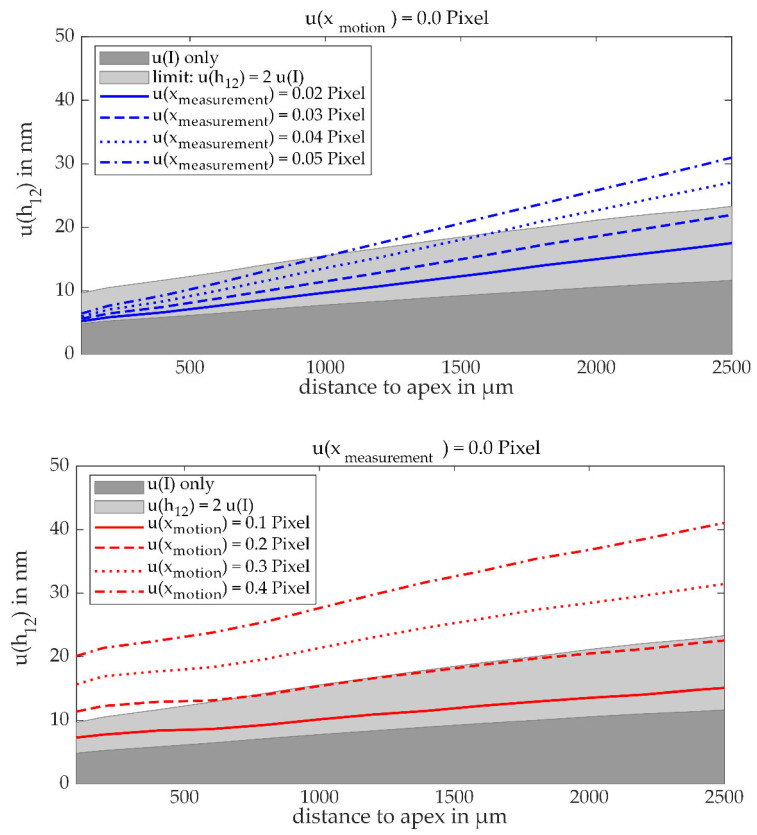
Resulting height uncertainty due to various levels of displacement measurement uncertainty and motion uncertainty. The dark grey area marks the uncertainty resulting from the intensity noise only. The light grey area marks the set limit of total height uncertainty equal to twice the intensity noise contribution. Top: The blue lines represent total height uncertainties, which are caused by varying amounts of displacement measurement uncertainty. Bottom: The red lines depict height uncertainties, which are caused by varying amounts of motion uncertainty.

The height uncertainty $u\left(h_{12}\right)$ calculated with the Monte Carlo simulation is presented in Figure 5 for different levels of the motion uncertainty $u\left(x_{\mathrm{motion}}\right)$ and the displacement measurement uncertainty $u\left(x_{\mathrm{measurement}}\right)$ over the distance of the field of view to the apex of the circular scan path. The top diagram in Figure 5 shows the influence of the displacement measurement uncertainty $u\left(x_{\mathrm{measurement}}\right)$ on the height uncertainty $u\left(h_{12}\right)$ without any motion uncertainty $u\left(x_{\mathrm{motion}}\right)$. The bottom graph shows the estimated height uncertainty for the opposite case, no $u\left(x_{\mathrm{measurement}}\right)$ but various levels of $u\left(x_{\mathrm{motion}}\right)$. For both simulated cases, the standard deviation of height increases with an increasing apex distance. As discussed in [11], the measurement uncertainty and the measurement range are connected in LSWLI. There is always a compromise to be made between the two, which is why measuring at a minimal apex distance with low height uncertainty is not always the optimal position for all measurement tasks, as sometimes a bigger measurement range is required.

The simulation results show that there is a lower uncertainty limit determined by image noise (dark grey in Figure 5), which in reality is unavoidable, but can be minimized by a careful setup of the optics and the camera. As stated in Section 1.2, a requirement for an appropriate displacement measurement system set for this article is that the total height uncertainty should not exceed the light grey areas in the figures, marking a total height uncertainty twice the intensity noise contribution. In the case presented at the top of Figure 5, showing the influence of the displacement measurement, the set uncertainty limit is only fulfilled for all considered apex distances with $u\left(x_{\mathrm{measurement}}\right)\;\leq \;0.02\;\mathrm{pixels}.$ Displacement measurement uncertainties beyond 0.05 pixels are only usable at very low apex distances, which are impracticable due to the low measuring range. The graph for $u\left(x_{\mathrm{measurement}}\right)\;=\;0.04\;\mathrm{pixels}$ intersects the upper border of the set uncertainty limit at an apex distance of 1.5 mm, which marks the upper limit for compromising the height uncertainty for measurement range in this article. In the case presented at the bottom of Figure 5, the same uncertainty limit can be fulfilled by height measurements with $u\left(x_{\mathrm{motion}}\right)\;\leq \;0.2\;\mathrm{pixels}$. Comparing the two simulated cases, it can be concluded that the displacement measurement uncertainty has a significantly larger impact on the height uncertainty than the motion uncertainty. Especially, it is essential that the proposed DSC displacement measurement system to be integrated in the LSWLI setup must have an uncertainty $u\left(x_{\mathrm{measurement}}\right)$ of significantly below 0.04 pixels.

### 3.2. Experiment

The enhanced LSWLI system with an integrated DSC displacement measurement system is tested on the measurement object introduced in Section 2.1. The sensor field of view is manually adjusted to an apex distance, which results in an interference region of 300 pixels length in scan direction. For the measured object, that means an apex distance of about 1.2 mm. In Section 3.2.1, the experimentally achieved displacement uncertainty of the DSC displacement measurement system is investigated. In Section 3.2.2, the obtained displacement values are applied to the LSWLI recordings in order to investigate the influence of the displacement measurement on the topography height uncertainty, enabling comparison between experiment and simulation.

#### 3.2.1. Displacement Measurement

The laser speckles of the DSC ROI were evaluated according to the method described in Section 2.1.1 to investigate the displacement measurement uncertainty u(x) achievable with the experimental rotatory LSWLI setup introduced in Section 2.1.3.

Before using the DSC measurement system as an aid for an actual LSWLI topography measurement, its capabilities were tested by carrying out standalone displacement measurements on a piezo linear stage with a linear position repeatability of ± 1 nm. For this, the WLI illumination was turned off, otherwise the setup of the LSWLI system is the same as in the topography measurement application. The stage was moved to 50 positions, spaced 557 nm (the size of the surface area imaged at a single pixel) apart, and at each position 100 DSC images were taken to statistically reduce the influence of vibrations. One hundred displacement measurement series were synthesized from the recorded 5000 images, whereby one measurement series consists of one displacement result for each of the 50 positions. The target displacement, which is 1 pixel for all displacements, was subtracted from all measured displacements, yielding the residual displacements for each single measurement. After filtering out outliers using an outlier-removal-algorithm applying the Grubbs test (based on [21]), the standard deviation of the residual displacements is calculated. It represents the empirical displacement measurement uncertainty of the DSC displacement measurement system, which reads $u\left(x_{\mathrm{measurement}}\right)=0.02\;\mathrm{pixel}$ (see Table 1). Converted to nm using the pixel size magnified through the setups optics yields $u\left(x_{\mathrm{measurement}}\right)=11.14\;\mathrm{nm}$, which seems low at this magnification, but is plausible since the interrogation windows used for correlation have a large size of 1200 x 100 pixels, which provides a lot of correlatable data to calculate statistically sound displacement values. Assuming no influence of $u\left(x_{\mathrm{motion}}\right)$ is present, the integrated DSC system works well enough to fulfil the set uncertainty limit according to the simulation results for rotatory LSWLI measurements at all simulated apex distances.

For topography measurements, a rotation stage for the movement of the measurement object is used, which has a unidirectional repeatability larger than that of the piezo linear stage. For these reasons, the measurement scan is subjected to both $u\left(x_{\mathrm{measurement}}\right)$ and $u\left(x_{\mathrm{motion}}\right)$. The motion uncertainty may even be increased if the object is not mounted perfectly centered, resulting in an unknown eccentric movement of the observed surface. Also, the object surface contains deviations such as waviness, which causes an apparent deviation in circumferential velocity due to local changes in object radius. The measured mean displacements are 0.9748 pixels/frame in x-direction and 0.003 pixels/frame in y-direction. As the y-displacement is well below the resolution of both the DSC system and the correlogram reconstruction algorithm, it is considered negligible for this study. Table 1 gives the standard deviation of displacement measurements for 3000 images taken for topography measurements. With a value of 0.26 pixels it is more than 10 times larger than the uncertainty obtained from the test on the piezo linear stage. As the illumination conditions of the DSC-ROI are the same in both DSC measurements, the displacement uncertainty of the rotatory LSWLI measurement can be mainly attributed to the influence of motion uncertainty.

The displacement uncertainty calculated for the rotatory LSWLI measurement is a result of both $u\left(x_{\mathrm{measurement}}\right)$ and $u\left(x_{\mathrm{motion}}\right)$. As the recording conditions for the DSC did not change, it is expected that $u\left(x_{\mathrm{measurement}}\right)\;=\;0.02\;\mathrm{pixels}$ is also the case in the rotatory LSWLI measurement. This marks the lower limit for the height uncertainty, reading $u{\left(h_{12}\right)}_{\mathrm{low}}\;=\;11\;\mathrm{nm}$ at the apex distance of 1.2 mm in the simulation (cf. Figure 5, top). A simulation with coupled uncertainties using the parameters$\;u\left(x_{\mathrm{measurement}}\right)\;=\;0.02\;\mathrm{pixels}$ in combination with $u\left(x_{\mathrm{motion}}\right)$ between 0.25–0.27 pixels at a distance of $x_{\mathrm{offset}}\;=\;1.2\;\mathrm{mm}$ yields an estimation for the height uncertainty in the range of $u\left(h_{12}\right)\;=\;21–24\;\mathrm{nm}$. This is outside the predefined uncertainty limit for the apex distance $x_{\mathrm{offset}}\;=\;1.2\;\mathrm{mm}$, which is $u{\left(h_{12}\right)}_{\mathrm{limit}}\;=\;16.7\;\mathrm{nm}$ according to the simulation. This means that the positioning conditions were not optimal in the experiment. As the height uncertainty of the measurement cannot be determined from the displacement measurements alone, an evaluation of the topography results will give insights into the height uncertainty achieved experimentally.

#### 3.2.2. Topography

**Figure 6:**
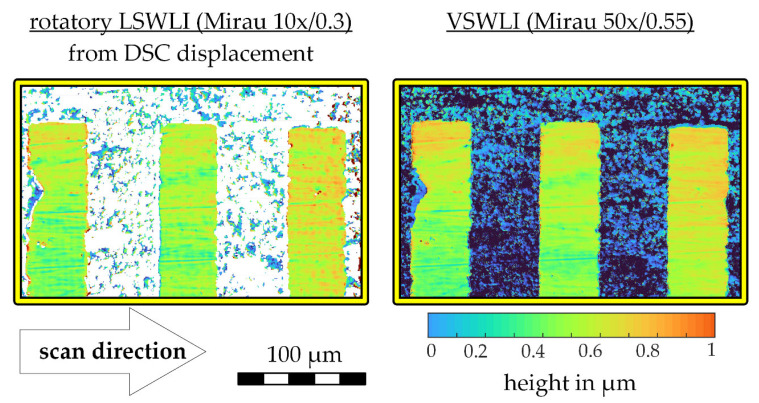
Results of topography measurements. **Left**: topography detail calculated using DSC displacements. **Right**: topography detail obtained from VSWLI reference measurement.

To showcase the influence of the displacement data on the calculation of the topography, the recording of the WLI ROI is evaluated with the DSC displacement data. The topography is evaluated with the same settings according to the methods described in Section 2. Figure 6 depicts a measured topography calculated using the DSC displacement measurement on the left and the topography of the same object recorded with the VSWLI with the 50× objective as a reference on the right. Note that the VSWLI topography shown in Figure 6 was captured without stitching. The curvature of the surface had to be subtracted from the VSWLI for the depiction in the right half of Figure 6 and the calculation following after. The curvature was removed using a parabolic fit over an averaged profile in scan direction. Additionally, LSWLI and VSWLI measurements were leveled using a linear fit over averaged profiles in both x- and y-direction.

In both resulting topographies, the characteristic features of the measurement object are visible. The object surface is made up of smooth stripes surrounded by a rougher region, which was manufactured with an etching process. While the VSWLI was able to capture both the rough and the smooth part, the LSWLI only provided topography data on the smooth part. This is due to the different measurement ranges of the two measurement devices. The VSWLI has a range of a few hundred µm and is able to capture both surface parts. The LSWLI has a range of about 15 µm and could therefore only capture the smooth regions. However, there are spots in the rough regions of the surface that could be evaluated. These are mainly regions, which were not completely eroded during the etching process. These small regions in the rough area of the object are characterized by their steep edges. Steep edges pose a challenge for WLI evaluation due to the lower amount of light scattered back into the WLI optics. For surface points at these edges, it is especially important to have a low displacement uncertainty to be able to reconstruct correlograms that are evaluable despite the lower light intensities received from these regions.

Finally, the rotatory LSWLI topography is quantitatively compared with a reference VSWLI measurement to assess the achieved height measurement uncertainty. As a figure of merit for the likeness between the results, the standard deviation of the height differences between the LSWLI result versus the VSWLI reference taken with a 50×/0.55 objective is calculated on the three smooth stripes visible in Figure 6. Additionally, the standard deviations, as a stand-in for the surface roughness parameter Sq, is calculated for eight of the smooth stripes, that were captured with the LSWLI with its 10×/0.3 objective and the VSWLI with a 10×/0.3 and a 50×/0.55 objective. At 50×, the topography was stitched with 40% overlap. The results are given in Table 2.

The obtained standard deviation of the height differences of the LSWLI measurement with the VSWLI reference is below the calculated surface roughness values, which implies that the LSWLI could be used for roughness measurements on this kind of surface. Another indication for the validity of the LSWLI system for this kind of smooth surface is that the confidence intervals of the LSWLI and both VSWLI measurements that were taken at different magnifications, are overlapping.

The LSWLI measurement was carried out about 1.2 mm away from the apex of the scan path, which, as derived in Section 3.2.1, should result in a height uncertainty of approx. $u\left(h_{12}\right)\;\approx \;23\;\mathrm{nm}$. For the topography obtained in the experiments, the height uncertainty is estimated with the height differences between rotatory LSWLI measurements and the VSWLI reference measurement, which was determined to be about $s\left({\Delta h}_{12}\right)\;=\;46\;\mathrm{nm}$, with a mean offset of $\bar{{\Delta h}_{12}}\;=\;0.011\;\mathrm{nm}$ achieved by manually aligning the two topographies. There are a number of reasons why the experimental height uncertainty is higher than estimated by the simulation.

Firstly, the VSWLI reference was recorded with a 50×/0.55 objective on a 0.5× tube, while the LSWLI measurement was carried out using a 10×/0.3 objective on a 1× tube. Even though the reference topography had to be downsampled to allow comparison with the LSWLI-based topography, its raw data was sampled with a higher lateral resolution and is therefore likely superior to the LSWLI regarding imaging of small surface features (<1 µm diameter) and steep edges, which affects the calculation of roughness. For the comparison itself, the two topographies were aligned manually with an alignment accuracy of ±0.5 pixel, which is based on the resolution of the pixel grid, which may be improved with a different sampling of the VSWLI reference topography to the grid.

Secondly, the recording perspectives of the LSWLI and the VSWLI differ. The LSWLI records the surface of the rotating object 1.2 mm away from the apex of the scan path, which means the surface is observed at an angle range of approx. 0.9–1.4°. The resulting topography of the rotatory LSWLI is a development of the specimen’s mantle surface, showing no curvature except the smaller scaled waviness and roughness of the surface. The VSWLI on the other hand records at a fixed position directly over the apex. Due to the curvature of the object’s surface, the topography is recorded including the cylindrical shape of the object, which has to be numerically removed, introducing deviations into the resulting VSWLI topography.

The aforementioned comparability issues of the rotatory LSWLI measurement results with the VSWLI reference topography may be explanations for the fact that the height uncertainty is worse than estimated by the simulation. Still, for an LSWLI setup in a developmental stage, on a real-world object with imperfections, (e.g. waviness, roughness) that were not considered in the simulations, the achieved height uncertainty of $u{\left(h_{12}\right)}_{\mathrm{real}}\;<\;50\;nm$ is considered a success.

## 4. Discussion

The article aimed to propose an integrated displacement measurement system for LSWLI that allows for topography measurements on continuously rotating objects. The influence of the uncertainty of the displacement measurement and the uncertainty of the scan motion was investigated by means of Monte Carlo simulation. The simulation showed that the displacement measurement uncertainty should not surpass 0.02 pixels to avoid doubling of the height uncertainty compared to a measurement system free of displacement uncertainty. The simulations showed further that the measurement of the position has to be about one order of magnitude more accurate than the lateral motion of the object itself.

The proposed integrated DSC system was tested in a standalone test on a piezo linear stage and in a rotatory LSWLI measurement. The standalone test under negligible influence of motion uncertainty revealed that the integrated DSC system is capable of displacement measurements with a sufficiently low uncertainty of $u\left(x_{\mathrm{measurement}}\right)\;=\;0.02\;\mathrm{pixels}$. At the 10× magnification used in this setup, this amounts to $u\left(x_{\mathrm{measurement}}\right)\;=\;11.14\;\mathrm{nm}$. As the DSC result is image-based, it can be assumed that at higher resolutions the DSC displacement measurement uncertainty will further decrease into the single nanometer range, provided the quality of the speckle pattern can be retained. In addition to the low measurement uncertainty and scalability to other magnifications, the integrated DSC displacement measurement system has further practical advantages as it works without calibration and requires no unit conversion or synchronization to the WLI recording, in contrast to, e.g., an external rotation encoder.

In rotatory LSWLI measurements, a total displacement uncertainty of u(x) = 0.26 pixels was observed. As the measurement circumstances of the DSC system were the same in the standalone test and the full topography measurement experiment, it is assumed that motion uncertainty is the dominant contributor to u(x) in the rotatory LSWLI measurement. The achieved total displacement uncertainty was not sufficient to fulfil the set uncertainty requirement. Compared to VSWLI topography measurement results, the LSWLI topography measurements have a higher height uncertainty, which is tolerated as LSWLI is intended to be used on continuously moving objects that cannot be measured with VSWLI. Accounting for the developmental status of the LSWLI setup compared to a commercial VSWLI and the difficult comparability of the flat LSWLI surface topography with the VSWLI topographies, which had to be flattened numerically and were recorded at a higher magnification, the calculated height uncertainty of the rotatory LSWLI system is considered plausible. The experimentally achieved height uncertainty is two times higher than estimated by the Monte Carlo simulation. A reason for this could be the difference between the perfectly smooth simulated surface and the rough real-world object. The influence of surface texture on the height uncertainty in rotatory LSWLI is a topic to be considered for future work. Further studies should be conducted to assess the repeatability of the topography measurement result during the recording of the surface over multiple rotations.

As the uncertainty of the integrated DSC displacement measurement is already sufficiently low, the most promising way to improve the height uncertainty is by reducing the influence of motion uncertainty. This issue could be tackled in future work by using superior rotation stages or by investigating strategies to compensate the negative effect of motion uncertainty during the evaluation process. Another field of study opened up by the integrated DSC system is improving the in-process capabilities and explore potential applications of the LSWLI technology. Having accurate information on the lateral location of the object may be used to tackle the issue of vibration in harsh industrial environments.

## Figures and Tables

**Table 1:** Standard deviations of digital speckle correlation (DSC) displacement measurements obtained from the rotatory lateral scanning white light interferometry (LSWLI) measurement and from the reference measurement on the linear stage.

Application	s in Pixel.
DSC linear stage test	0.02
DSC in rotatory LSWLI	0.26

**Table 2:** Standard deviations of the height differences between LSWLI topography and vertical scanning white light interferometry (VSWLI) reference topography. The standard deviation of each topography by itself is given as a figure of merit for the surface roughness.

**Height Differences**	**s(Δh_12_) in nm**
DSC - reference	46.2
**surface roughness**	**Sq in nm**
LSWLI + DSC	55.9 ± 5.3
Reference 50×/0.55	57.5 ± 6.9
Reference 10×/0.3	52.5 ± 10.7

## Data Availability

Data will be made available on request.
